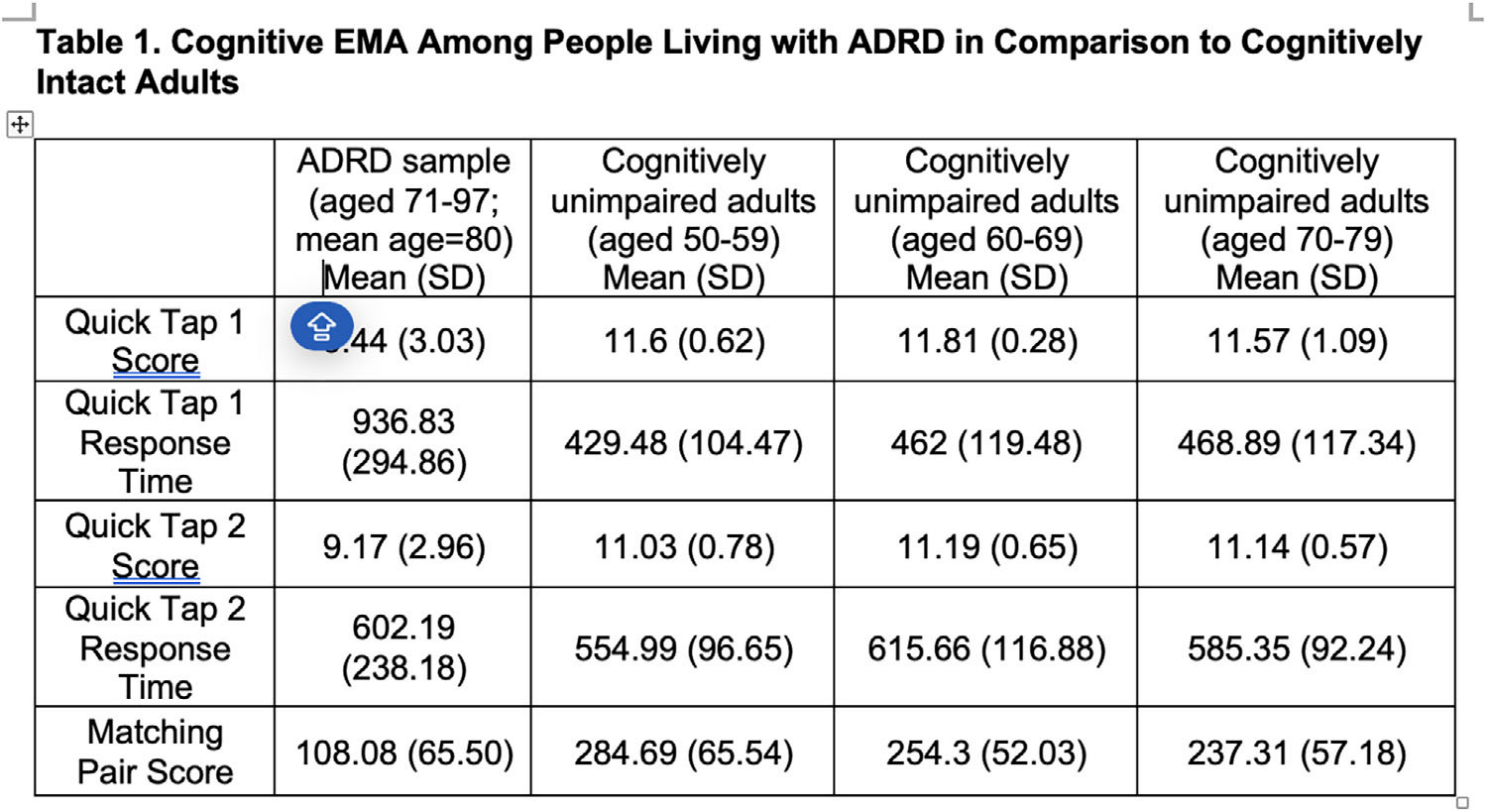# Feasibility of Using Smartphone‐Based Cognitive Testing Among People Living with Alzheimer’s Disease and Related Dementias

**DOI:** 10.1002/alz.091422

**Published:** 2025-01-03

**Authors:** Raeanne C Moore, Kevin Kuehn, Diviya Khullar, Carissa Jantz, Jessie M Song, Prasanna Padmanabham, Brent Mausbach, Jennifer L Martin, Yeonsu Song

**Affiliations:** ^1^ University of California San Diego, La Jolla, CA USA; ^2^ University of California Los Angeles, Los Angeles, CA USA; ^3^ VA Greater Los Angeles Healthcare System, North Hills, CA USA

## Abstract

**Background:**

Unsupervised high‐frequency cognitive‐ecological momentary assessment (EMA) on smartphones is increasingly used to assess preclinical risk for Alzheimer’s disease and related dementias (ADRD). While these tests are reliable and valid for individuals with preclinical ADRD, it is unclear whether administering cognitive EMAs to people living with ADRD is feasible and valid. Our study explored the feasibility and validity on cognitive EMA tests in people living with moderate to severe ADRD.

**Method:**

A total of 31 with ADRD received cognitive EMA web links through their care partners' smartphone three times/day for seven days at baseline as part of an ongoing clinical trial of a dyadic sleep intervention. Of which, 23 completed any of three cognitive EMA tests using the NeuroUX platform (Quick Tap 1 [QT1], Quick Tap 2 [QT2], Matching Pair [MP]). We examined baseline adherence to cognitive EMA prompts, performance distributions, and within‐day variability of cognitive performance. We also compared this data to a normative sample.

**Result:**

On average, participants completed 60% of cognitive EMAs (SD = 27%). This was lower than a normative sample of cognitively normal adults aged 20‐79 (N = 394; mean adherence = 88%, SD = 21%). Participants tapped the response correctly 78.68% (SD = 0.25%) of the time on QT1, a measure of psychomotor speed, and took an average of 936.83 milliseconds (SD = 294.86) to respond correctly (see Table 1 for comparison to normative data). On a measure of response inhibition (QT2), participants responded correctly on 33.07% of the items (SD = 0.19%), with mean correct response times of 602.19 milliseconds (SD = 238.18). Finally, participants scored a mean of 108.08 (SD = 65.60) on MP, a measure of processing speed. Performance on QT2 and MP (% of correct response) did not differ throughout the day; however, participants response times to QT2 was significantly slower in the morning than in the afternoon or evening (morning *M* = 643.81; afternoon *M* = 587.14; evening *M* = 578.19; *F*(df = 1) = 7.24, *p* < 0.01).

**Conclusion:**

Current findings extend research on people with preclinical ADRD, demonstrating that reliable and valid cognitive EMA data can be obtained from this group and employed in research.